# An Uncommon Presentation of Leptomeningeal Metastases in Breast Carcinoma Detected by F-18 FDG PET/CT

**DOI:** 10.1055/s-0042-1757254

**Published:** 2022-09-09

**Authors:** Gavini Surya, Nimmagadda Ajit, Rallapeta Ramya Priya, Dhamarcherla S. Hemalatha, Bodagala Vijayalakshmi Devi, Tekchand Kalawat

**Affiliations:** 1 Department of Nuclear Medicine, Sri Venkateswara Institute of Medical Sciences, Tirupati, Andhra Pradesh, India; 2 Department of Radiology, Sri Venkateswara Institute of Medical Sciences, Tirupati, Andhra Pradesh, India

**Keywords:** breast cancer, F-18 FDG PET/CT, leptomeningeal metastases

## Abstract

Leptomeningeal carcinomatosis is a manifestation in which tumor cells migrate into meninges. Breast carcinoma presenting with leptomeningeal metastases is a rare phenomenon that can occur in an isolated form as well as with coexistent parenchymal brain metastases. The gold standard for diagnosis is cerebrospinal fluid analysis, while contrast-enhanced magnetic resonance imaging is the most commonly used imaging modality. Nuclear medicine imaging with flourine-18-fluorodeoxyglucose positron emission tomography/computed tomography has proved to be useful in detecting leptomeningeal metastases and, at times, even before anatomical changes occur. Here, we present a case of breast carcinoma presenting with both pachymeningeal and leptomeningeal metastases 10 years after treatment.

## Introduction

Leptomeningeal carcinomatosis is a late-stage manifestation of systemic cancers where tumor cells invade leptomeninges containing pia, arachnoid matter, and subarachnoid space. It can occur through direct invasion, venous dissemination, hematogenous spread, or through neural route. 1 Leptomeningeal metastases are commonly associated with breast cancer, lung cancer, and malignant melanoma. Rarely, it is also seen in certain gastrointestinal, primary brain tumors, and hematological malignancies. 2

Patients generally present with clinical features related to increase in intracranial pressure and cerebrospinal fluid (CSF) obstruction such as headache, nausea, vomiting, radiculopathy, and decreased level of consciousness. CSF analysis is considered as the gold standard for diagnosis. Magnetic resonance imaging (MRI) is indicated in clinically suspicious cases and with negative cytology where it can detect nodularity and leptomeningeal enhancement. CSF flow studies by indium-111 (In-111) DTPA can sometimes be useful in detection of obstruction of CSF flow. Flourine-18-fluorodeoxyglucose positron emission tomography computed tomography (F-18 FDG PET/CT) is known to help in detection of leptomeningeal involvement of both intracranial and extracranial structures, as in this case report of a carcinoma right breast with leptomeningeal metastases.

## Case Report

A 65-year-old female patient with invasive lobular carcinoma of right breast underwent right modified radical mastectomy, and adjuvant chemotherapy with six cycles of paclitaxel and carboplatin and subsequently remained event free for 6 years; later she was diagnosed with skeletal metastases and underwent palliative radiotherapy and was maintained on hormonal therapy with tab. tamoxifen for 4 years. Later, she complained headache and giddiness without associated nausea or loss of consciousness and was evaluated for the same.

Contrast-enhanced MRI using T1-weighted and T2-weighted sequences was performed that revealed nodular thickening along with pachy- and leptomeningeal enhancement in bilateral cerebral hemispheres and bilateral cerebellar regions in concurrent with cerebral metastases in right frontal cortex.

**Fig. 1:**
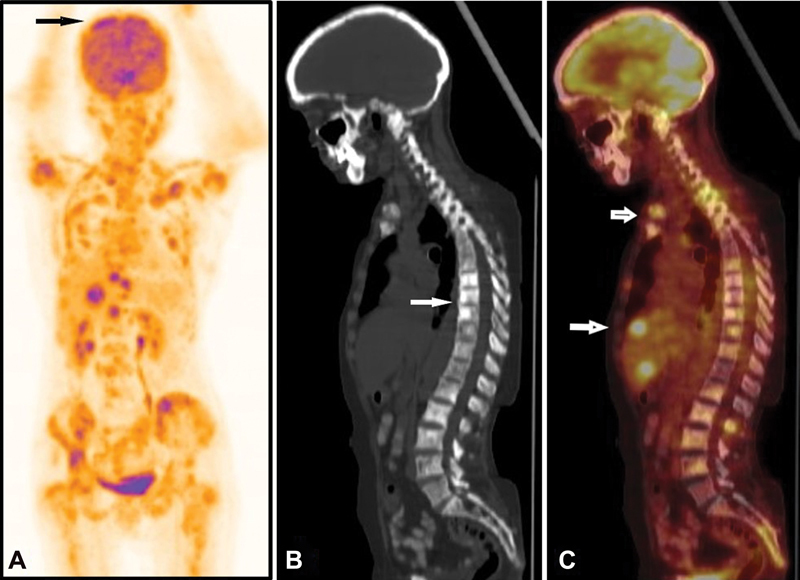
F-18 FDG PET CT maximum intensity projection (MIP) image ( **A** ) and sagittal CT ( **B** ) and fused PET CT ( **C** ) images showing no abnormal metabolic activity at primary treated site and moderately increased FDG concentration at leptomeninges, liver and multiple sclerotic lesions in skeletal system.

**Fig. 2:**
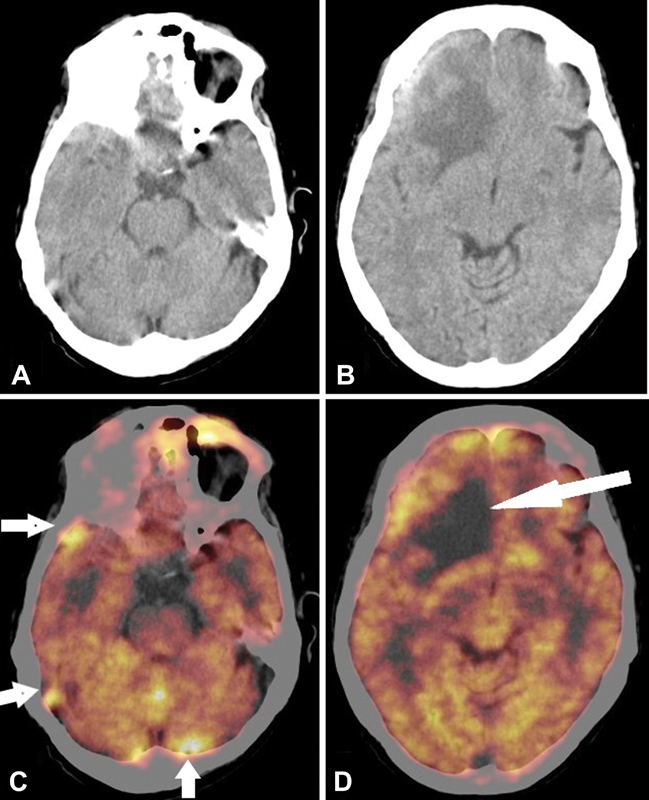
Transverse CT ( **A, B** ) and fused PET CT ( **C, D** ) images showing moderately increased FDG concentration in leptomeninges of right frontal, temporal cortex and bilateral cerebellar regions along with parenchymal lesion in right frontal cortex with disproportionate edema.

One week later, F-18 FDG PET/CT was performed that showed widespread multiple FDG-avid sclerotic bone lesions in vertebral column, ribs, long bones and metabolically active hypodense lesions in both lobes of liver along with mediastinal lymphadenopathy ( Fig. 1 ). Brain images showed isodense area of non-FDG-avid lesion in right frontal cortex with surrounding diffuse disproportionate edema and mass effect on right side basal ganglia and anterior horn of right lateral ventricle. There are multiple focal and diffuse leptomeningeal thickening with increased metabolic activity in bilateral cerebral hemispheres, predominantly in right frontal, left parietal convexity, and bilateral cerebellar regions ( Fig. 2 ). All findings are suggestive of progressive disease with late sequela of brain parenchymal and lepto- and pachymeningeal deposits.

## Discussion

Leptomeningeal metastases in breast cancer are a rare presentation that constitute up to 3 to 5% of overall metastases. 3 Lobular type of breast carcinoma has more propensity for leptomeningeal metastases than others. 4 The deficiency of E-cadherin complex, a crucial factor for maintenance of normal cytoarchitecture, is a favorable factor for metastases invasion. 5 Triple-negative subtype of breast carcinoma constitutes major proportion in those progressing to leptomeningeal metastases and tends to decrease median time of onset of metastases from onset of primary. 6 Existing bone metastases particularly vertebral and paravertebral lesions can infiltrate leptomeninges by spreading along perivascular spaces of venous plexus .

Most common clinical feature is headache and other features are focal neurological deficits, visual disturbances, diplopia, and radiculopathy depending on neuronal structure involved by malignant cells; these cells can grow and obstruct the flow of CSF leading to symptoms of obstructive hydrocephalus such as nausea, vomiting, and positional headaches. 7

CSF examination can demonstrate tumor cells has sensitivity of up to 90% after three successive lumbar punctures. 8 Other nonspecific parameters include elevation of biomarkers and cytokines, elevated CSF pressure, increased protein levels, leukocytosis, and decreased glucose levels. 9 However, in our case, the patient did not undergo CSF analysis.

MRI with gadolinium contrast is more sensitive than CT and should be performed in patients with clinical suspicion and should cover entire brain and spinal cord, as leptomeningeal carcinomatosis can involve entire neuronal axis. Pial enhancement and nodularity can involve cerebral convexities, tentorium, ventricular ependymal surfaces, and cauda equina. 10 CSF flow studies by In-111 DTPA can detect site of obstruction and provide guide for focused radiotherapy. 11

F-18 FDG PET/CT imaging is a known as an efficient modality in detecting metastases. Also, whole-body survey for primary tumor, local recurrence, metastases, and evaluation of treatment response can be done in a single sitting. However, it has certain limitations in structures with high background activity such as brain. In literature, several reports suggested the utility of F-18 FDG PET/CT in detection of both intra- and extracranial leptomeningeal metastases. 12 13

Short et al reported a case of leptomeningeal metastases, where lesions were seen on F-18 FDG PET/CT with normal MRI findings. 14 In our case, F-18 FDG PET/CT not only detected leptomeningeal metastases but also detected brain, liver, and skeletal metastases along with mediastinal lymphadenopathy.

Treatment options for leptomeningeal metastases include intrathecal radiotherapy, radiotherapy, and systemic chemotherapy. 15 Despite all the treatment modalities available, leptomeningeal metastases in breast carcinoma have a poor prognosis with median survival time of 3.5 months and 1 year survival rate is almost 20%. 16

## Conclusion

Leptomeningeal metastases are a late and rare presentation in breast carcinoma with poor prognosis. Even though early detection may not significantly improve survival, it can help in planning of palliative management. As illustrated in our case report, F-18 FDG PET/CT detected leptomeningeal metastases along with other metastatic lesions in brain, skeleton, and liver.
